# Targeting Sirtuin 1 for therapeutic potential: Drug repurposing approach integrating docking and molecular dynamics simulations

**DOI:** 10.1371/journal.pone.0293185

**Published:** 2023-12-20

**Authors:** Mohammed Alrouji, Fahad A. Alhumaydhi, Abdulrhman Alsayari, Sharaf E. Sharaf, Sheeba Shafi, Saleha Anwar, Moyad Shahwan, Akhtar Atiya, Anas Shamsi

**Affiliations:** 1 Department of Medical Laboratories, College of Applied Medical Sciences, Shaqra University, Shaqra, Saudi Arabia; 2 Department of Medical Laboratories, College of Applied Medical Sciences, Qassim University, Buraydah, Saudi Arabia; 3 Department of Pharmacognosy, College of Pharmacy, King Khalid University (KKU), Abha, Saudi Arabia; 4 Complementary and Alternative Medicine Unit, King Khalid University (KKU), Abha, Saudi Arabia; 5 Pharmaceutical Chemistry Department, College of Pharmacy Umm Al-Qura University Makkah, Makkah, Saudi Arabia; 6 Department of Nursing, College of Applied Medical Sciences, King Faisal University, Al-Ahsa, Saudi Arabia; 7 Centre for Interdisciplinary Research in Basic Sciences, Jamia Millia Islamia, New Delhi, India; 8 Center for Medical and Bio-Allied Health Sciences, Ajman University, Ajman, UAE; Army Medical University, CHINA

## Abstract

Identifying novel therapeutic agents is a fundamental challenge in contemporary drug development, especially in the context of complex diseases like cancer, neurodegenerative disorders, and metabolic syndromes. Here, we present a comprehensive computational study to identify potential inhibitors of SIRT1 (Sirtuin 1), a critical protein involved in various cellular processes and disease pathways. Leveraging the concept of drug repurposing, we employed a multifaceted approach that integrates molecular docking and molecular dynamics (MD) simulations to predict the binding affinities and dynamic behavior of a diverse set of FDA-approved drugs from DrugBank against the SIRT1. Initially, compounds were shortlisted based on their binding affinities and interaction analyses to identify safe and promising binding partners for SIRT1. Among these candidates, Doxercalciferol and Timiperone emerged as potential candidates, displaying notable affinity, efficiency, and specificity towards the binding pocket of SIRT1. Extensive evaluation revealed that these identified compounds boast a range of favorable biological properties and prefer binding to the active site of SIRT1. To delve deeper into the interactions, all-atom MD simulations were conducted for 500 nanoseconds (ns). These simulations assessed the conformational dynamics, stability, and interaction mechanism of the SIRT1-Doxercalciferol and SIRT1-Timiperone complexes. The MD simulations illustrated that the SIRT1-Doxercalciferol and SIRT1-Timiperone complexes maintain stability over a 500 ns trajectory. These insightful outcomes propose that Doxercalciferol and Timiperone hold promise as viable scaffolds for developing potential SIRT1 inhibitors, with implications for tackling complex diseases such as cancer, neurodegenerative disorders, and metabolic syndromes.

## 1. Introduction

Identifying novel therapeutic agents to combat a spectrum of diseases, ranging from cancer and neurodegenerative disorders to metabolic syndromes, is a formidable challenge in modern drug discovery [1]. The complexities of these diseases often stem from the dysregulation of complex molecular pathways, necessitating a multifaceted approach that encompasses a deep understanding of the underlying molecular mechanisms [2]. As these diseases’ biological and biochemical complexities continue to be unveiled, various innovative drug discovery approaches emerged over the years [3]. One such approach gaining significant attention is drug repurposing–the concept of repositioning existing drugs originally developed for one therapeutic purpose into novel applications [4]. This strategy presents an avenue for expedited drug development, avoiding the lengthy timelines and extensive costs associated with *de novo* drug design and development [5]. Moreover, repurposing existing drugs capitalizes on the extensive knowledge about their safety profiles and accelerates the translation of research findings into substantial clinical benefits [6].

This study comprehensively explored drug repurposing approach targeting the Sirtuin 1 (SIRT1) protein. SIRT1, a member of the sirtuin family of NAD^+^-dependent histone deacetylases, has emerged as an important protein with a multifaceted role in numerous cellular processes [7]. With its involvement in various physiological pathways, including metabolism, DNA repair, apoptosis, and inflammation, SIRT1 has gotten considerable attention as a potential therapeutic target for various diseases [8]. Among these, cancer, neurodegenerative disorders such as Alzheimer’s, Huntington’s and Parkinson’s diseases, as well as metabolic syndromes including obesity and diabetes, stand out as areas of substantial interest [7, 9, 10]. SIRT1 comprises three major structured regions: the N-terminal region spanning residues 183–229, the catalytic core region encompassing residues 229–516, and a distant region around 641–665 known as the C-terminal regulatory segment (CTR) peptide [11]. Residues 183–229 within the N-terminal region of SIRT1 play a pivotal role in binding sirtuin–activating compounds (STACs). Meanwhile, substrate binding takes place within the catalytic domain of SIRT1, which consists of a large NAD_+_ binding Rossmann-fold domain and a smaller zinc-binding domain [12].

SIRT1 plays a dual role in disease progression [13, 14]. Its function varies across different stages of tumorigenesis, depending upon tissue context and cancer type. Furthermore, SIRT1 can exhibit distinct expression patterns at various stages of tumor development [13]. Clinically, elevated SIRT1 expression has been associated with relapsed patients, and those with high SIRT1 expression tend to have poorer outcomes [15]. Studies have delineated the multifaceted role of SIRT1 in different stages of cancer, including its involvement in genome instability, tumor initiation, proliferation, metabolism, and therapeutic responses [13]. Consequently, SIRT1 inhibitors have exhibited promising anti-cancer effects in animal models of cancer [16]. There are a few SIRT1 inhibitors known such as, Cambinol analogs [17], Sirtinol [18], and Selisistat [19]. Clinical trials have demonstrated that Sirt1 inhibition by Selisistat can restore transcriptional dysregulation in models of Huntington’s disease [14]. The critical regulatory functions of SIRT1 in these diseases highlight its potential as a pharmacological target, prompting efforts to identify selective and potent inhibitors that can modulate its aberrant activity [10].

Historically, drug discovery has often been driven by the search for compounds with novel chemical structures and mechanisms of action [20]. However, this approach can be hampered by high failure rates, lengthy development timelines, and mounting costs. As a response to these challenges, drug repurposing has emerged as a reasonable alternative [21]. Repurposing leverages existing knowledge about approved drugs’ safety, pharmacokinetics, and pharmacodynamics and repurposes them for new indications, thereby accelerating the translation of promising compounds into clinical practice [6]. By harnessing this approach, we can optimize the use of existing resources in terms of data and compounds to address unmet medical needs more swiftly.

The advent of computational biology and bioinformatics has dramatically transformed the drug discovery landscape [22]. Integrating *in silico* techniques, such as molecular docking and molecular dynamics (MD) simulations, has paved the way for rational and efficient drug design strategies [23]. Molecular docking facilitates the prediction of the binding interactions between a protein target and small molecule ligands, thereby enabling the identification of potential drug candidates [24]. Subsequently, MD simulations enable us to investigate the dynamic behavior and stability of protein-ligand complexes at an atomistic level, providing insights into the conformational changes and binding mechanisms that underlie their interactions [25]. When applied synergistically, molecular docking and MD simulations offer a powerful platform for virtual screening and lead optimization, considerably expediting the drug discovery pipeline [26].

In the context of SIRT1 inhibition, this study seeks to bridge the gap between computational methodologies and therapeutic potential. We aim to identify and evaluate promising FDA-approved drugs that could serve as SIRT1 inhibitors by employing a systematic and comprehensive bioinformatics approach. Using repurposed drugs as potential inhibitors offers a unique advantage by tapping into their established safety profiles, potentially reducing the barriers associated with drug development. Our approach integrates molecular docking and MD simulations to predict binding affinities, interaction patterns, and dynamic behaviors of a diverse range of FDA-approved drugs against the SIRT1 protein. This strategy aims to uncover candidate compounds with the propensity to bind to the active site of SIRT1 and inhibit its activity, thereby modulating disease-relevant pathways.

The initial compounds were shortlisted based on binding affinities and interaction analyses. Among these, Doxercalciferol and Timiperone emerge as a promising candidate, demonstrating remarkable affinity, efficiency, and specificity for the SIRT1 binding pocket. We then proceed to an in-depth evaluation of Doxercalciferol and Timiperone’s potential as SIRT1 inhibitors, emphasizing their favorable biological properties and preference for binding to the active site of SIRT1. We employed all-atom MD simulations to unravel the dynamic behavior of the SIRT1-Doxercalciferol and SIRT1-Timiperone complexes, which reveal the complex’s stability and interaction mechanisms over an extended trajectory of 500 nanoseconds (ns). Our findings not only shed light on the binding characteristics of Doxercalciferol and Timiperone but also underline the utility of computational approaches in drug repurposing. Ultimately, our study presents Doxercalciferol and Timiperone as promising scaffolds for developing potential SIRT1 inhibitors, offering implications for therapeutic interventions in complex diseases.

## 2. Material and methods

### 2.1 Data preparation: SIRT1 structure and FDA-approved drugs database

An established computational workflow for drug design and discovery was employed, utilizing various bioinformatics tools, including MGL AutoDock Tools [27], AutoDock Vina [28], Discovery Studio Visualizer [29], and GROMACS [30], to conduct virtual screening (VS) and MD simulations. The three-dimensional crystal structure of the human SIRT1 protein was retrieved from the Protein Data Bank (PDB ID: 4I5I) [31]. Before the docking study, the protein structure was checked for missing atoms, solvent compounds, and heteroatoms. The protein structure was then prepared by removing water molecules and other non-essential components. The energy minimization of the SIRT1 structure was performed in Swiss-PDB-Viewer tool [32]. Hydrogens were added, and protonation states were adjusted based on the physiological pH using MGL AutoDock tools. A comprehensive dataset of 3648 FDA-approved drugs was sourced from DrugBank [33]. This dataset was filtered to exclude biologics, peptides, and other non-small molecule compounds, resulting in a curated collection of small molecule FDA-approved drugs. Each drug molecule was retrieved in SDF 3D structure for subsequent molecular docking. The 3D structures of the FDA-approved drug compounds were subjected to energy minimization and geometry optimization using GROMOS 43B1 force field [34]. The MGL AutoDock tools were utilized for ligand preparation. The prepared ligands were then subjected to ionization state prediction and tautomeric enumeration, ensuring an accurate representation of the potential ligand conformations.

### 2.2 Molecular docking screening

We performed a molecular docking-based virtual screening (VS) to filter compounds based on their binding affinities towards SIRT1. Molecular docking studies were performed using AutoDock Vina, a widely utilized docking program. The protein structure was prepared by adding polar hydrogens and assigning charges. The grid was generated around the whole SIRT1 using blind dimensions. The grid dimensions for the X, Y, and Z coordinates were defined as follows: X-axis = 51 Å, Y-axis = 83 Å, and Z-axis = 65 Å. The central point of this grid was established at specific coordinates: X-axis: 41.138 Å, Y-axis: −25.622 Å, and Z-axis: 22.673 Å. A uniform grid spacing of 1 Å was employed. All other parameters related to the molecular docking procedure were maintained at their default settings. Vina standard VS protocol was employed initially to screen the entire FDA-approved drug dataset. The robustness of the docking protocol was rigorously confirmed using a redocking approach, a well-established and extensively documented technique in the scientific literature [35]. Specifically, the validation process of the Vina-default docking protocol entailed a comprehensive assessment through retrospective docking. This assessment involved redocking a co-crystalized reference inhibitor (Selisistat analogue, (6S)-2-chloro-5,6,7,8,9,10-hexahydrocyclohepta[b]indole-6-carboxamide) with SIRT1, followed by a comparison between docked and co-crystalized poses.

The results of this validation step unequivocally demonstrated the precise and reliable performance of our docking protocol. Notably, the Selisistat analogue, when subjected to our docking methodology, consistently predicts a binding pose that corresponded precisely to the location of the co-crystallized Selisistat analogue within the SIRT1 structure (PDB ID: 4I5I). This convergence of binding poses was visually depicted in **S1 Fig**, where the binding configurations of the docked Selisistat analogue and the crystallographically determined Selisistat analogue were superimposed. In essence, the superimposition of the docked and co-crystallized Selisistat analogues’ binding poses serves as compelling evidence of the accuracy and effectiveness of our docking protocol. This validation procedure, underpinned by a redocking strategy, reinforces the reliability of our approach and underscores its potential utility in guiding further studies and applications within the realm of molecular docking and ligand interaction analysis.

### 2.3 Scoring and interaction analysis

The docking results were analyzed based on their Vina score and ligand efficiency, which combines multiple energy terms to predict binding affinities. Subsequently, interaction analysis was carried out on the top-ranked compounds from VS. The top-ranking hit candidates were visually inspected for binding interactions with critical amino acid residues within the SIRT1 binding pocket using PyMOL and Discovery Studio molecular visualization software. Based on the molecular docking and interaction analyses, a subset of FDA-approved drugs displaying favorable binding affinities and interactions with SIRT1 was shortlisted. The shortlisted compounds were then evaluated for various pharmacological properties in PASS analysis for their potential against SIRT1 associated diseases.

### 2.4 PASS analysis: Biological activity predictions

The potential biological characteristics of the chosen compounds were explored utilizing the PASS web server [36]. This tool enables an investigation into the plausible biological attributes of compounds, relying on their chemical composition. It employs 2D molecular fragments called multilevel neighbors of atoms (MNA) descriptors, which indicate that the biological effect of a chemical compound is linked to its molecular arrangement. The tool provides a predictive score for biological traits based on the ratio of ’probability to be active (Pa)’ and ’probability to be inactive (Pi)’. A higher Pa value indicates a greater likelihood of compounds exhibiting a specific biological trait. In this context, the focus was on interactions with SIRT1 and biological features indicative of SIRT1-inhibitory potential. Ultimately, two compounds, Doxercalciferol and Timiperone were chosen for MD simulation investigations.

### 2.5 MD simulations

Three distinct systems were subjected to all-atom MD simulations: the unbound SIRT1 and the SIRT1-Doxercalciferol and SIRT1-Timiperone complexes. These simulations were conducted at 300 K using the GROMOS 54A7 force field within the GROMACS v5.1.2 simulation package [30]. The molecular topology parameters for Doxercalciferol and Timiperone were derived from the ATB server [37] and integrated into the protein topology to form the SIRT1-Doxercalciferol and SIRT1-Timiperone complexes. Subsequently, the complexes were immersed in a pre-equilibrated periodic water box, with counterions added to maintain system neutrality. The free SIRT1, SIRT1-Doxercalciferol and SIRT1-Timiperone systems were enveloped in cubic boxes, utilizing the SPC (spc216) water model for aqueous environments [38]. Energy minimization involving 1500 steps of the steepest descent method was executed for 1000 ps. During equilibration, the system temperature was gradually raised from 0 to 300 K. This equilibration phase spanned 1000 ps, maintaining constant volume under periodic boundary conditions and a stable pressure of 1 bar. For the final MD run, all three systems underwent a 500 ns simulation, and the ensuing trajectories were subject to analysis through GROMACS’ integrated tools. Trajectory analysis encompassed the evaluation of the SIRT1-ligands complex’s stability across the simulation duration. Systematic parameter calculations were conducted to monitor and quantify the structural changes and fluctuations occurring within the complex.

### 2.6 Principal component analysis

To investigate conformational variability, atomic dynamics, and structural robustness of SIRT1, the SIRT1-Doxercalciferol and SIRT1-Timiperone complexes, we conducted principal component analysis (PCA) using the essential dynamics methodology, which involves the computation of the covariance matrix [39]. The covariance matrix was computed using the following formula:

$$C_{ij}=<\left(x_{i}‐<x_{i}>)(x_{j}‐<x_{j}>\right)>$$

where x_i_/x_j_ is the coordinate of the i^th^/j^th^ atom of the system, and <—> is the ensemble average.

### 2.7 Free energy landscape analysis

The free energy landscapes (FELs) for SIRT1, the SIRT1-Doxercalciferol and SIRT1-Timiperone complexes were constructed through a conformational sampling technique, enabling the exploration of protein conformations in the locality of their native states. These FELs were constructed to analyze the stability and native configurations of SIRT1, both prior to and after the binding of Doxercalciferol and Timiperone. The generation of FELs involved the utilization of the subsequent formula:

$$\Delta G(X)=‐K_{B}T\ln\;P(X)$$

where *K*_B_ is the Boltzmann constant, *T* is the simulation temperature, and *P*(X) is the probability distribution of the system along with the PCs.

## 3. Results

### 3.1 Molecular docking screening

Our study commenced with an extensive molecular docking investigation involving a diverse collection of 3648 FDA-approved small molecule drugs against the SIRT1 protein. Employing a robust VS protocol, we efficiently sifted through the entire dataset, pinpointing a subset of compounds displaying promising binding affinities for SIRT1. Subsequently, we evaluated the ligand efficiency of the top-ranked compounds to identify more effective binding partners for SIRT1. From the VS analysis, 10 compounds emerged as standout candidates from a pool of 3648 compounds, exhibiting notable binding scores ranging from −9.8 kcal/mol to −10.5 kcal/mol towards SIRT1 (**Table 1**). Notably, all these hits exhibited a greater affinity for SIRT1 compared to the reference inhibitor, Selisistat, which recorded a calculated binding score of −8.1 kcal/mol. This compelling result suggests that the identified compounds have the potential to serve as superior binding partners for SIRT1, making them promising candidates for further investigation as SIRT1 inhibitors to modulate its activity.

**Table 1:** List of selected hit molecules and their docking score.

S. No.	Drug	Binding Affinity (kcal/mol)	Ligand Efficiency (kcal/mol/non-H atom)
1.	Nilotinib	−10.5	0.27
2.	Dihydrotachysterol	−10.3	0.36
3.	Pyrvinium	−10.1	0.35
4.	Lycopene	−10.1	0.25
5.	Lapatinib	−10.0	0.25
6.	Benzquercin	−10.0	0.18
7.	Irinotecan	−9.9	0.23
8.	Dihydroergocristine	−9.8	0.22
9.	Timiperone	−9.8	0.35
10.	Doxercalciferol	−9.8	0.33
11.	Selisistat	−8.1	0.47

### 3.2 Interaction analysis

The interaction patterns of the selected 10 hits from the docking study were meticulously analyzed to shortlist promising candidates for further evaluation. An in-depth interaction analysis of these leading candidates was carried out utilizing PyMOL and Discovery Studio Visualizer. Among these high-scoring compounds, our selection process was guided by binding affinities and interactions within SIRT1’s active site. During this process, we extracted all possible docked conformations from the output files of the chosen hits and the SIRT1 reference inhibitor, Selisistat. The binding poses specifically interacting with the active site residues of SIRT1 were selected for the analysis. Here, compounds Doxercalciferol and Timiperone consistently emerged as standout candidates, forming specific hydrogen bonds and hydrophobic interactions with critical residues within the binding pocket of SIRT1.

**Fig 1:**
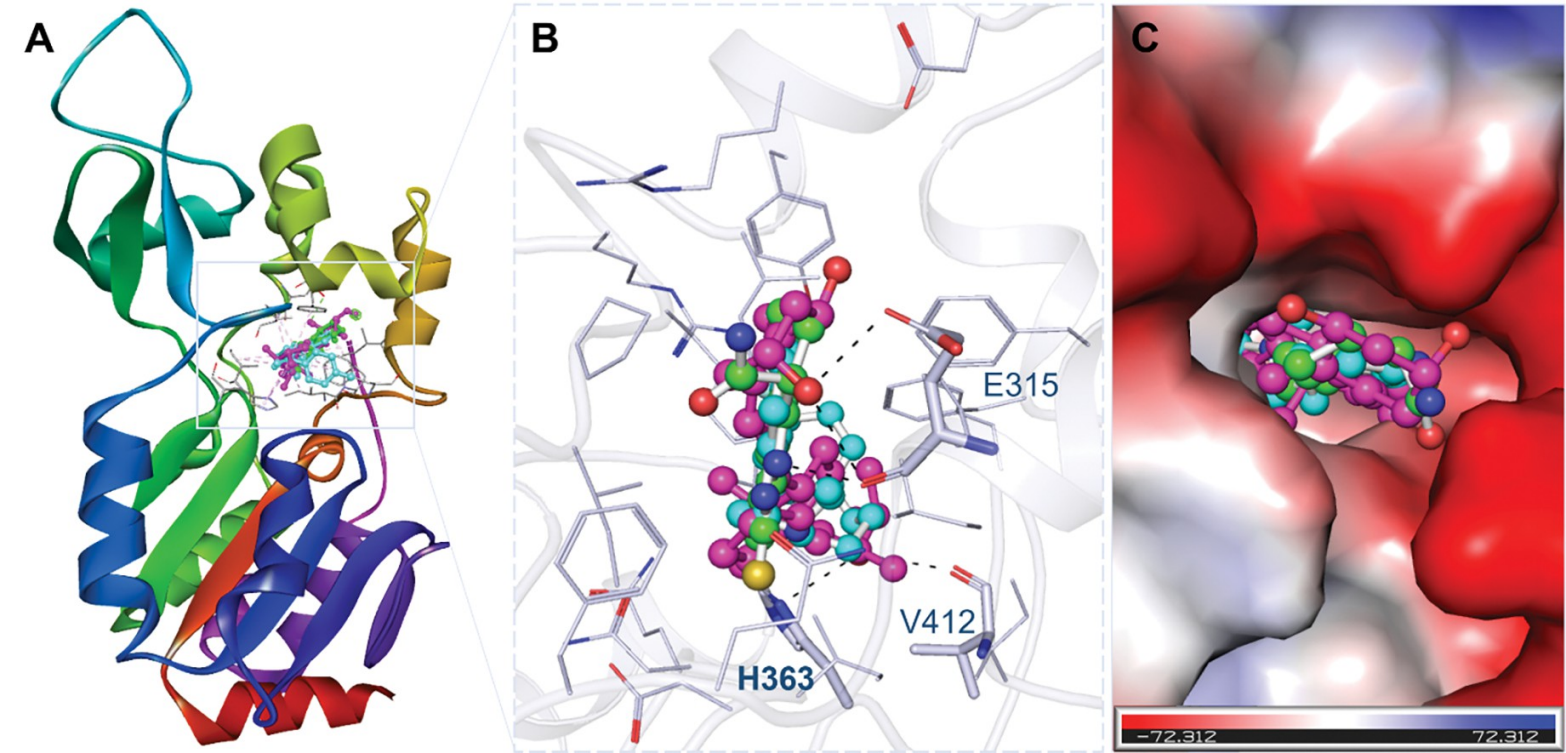
Structural representation of docked compounds in the binding pocket of SIRT1. (**A**) Cartoon representation of SIRT1 with Doxercalciferol (magenta) and Timiperone (cyan) along with the reference inhibitor Selisistat (green). **(B)** Surface potential view of SIRT1 binding pocket occupied by the selected compounds and Selisistat.

Through a comprehensive assessment of all conceivable docked conformers, we chose Doxercalciferol and Timiperone, that preferentially bind with SIRT1’s active site, i.e., His363 **(Fig 1)**. The binding patterns of Doxercalciferol and Timiperone with SIRT1 are visually depicted in **Fig 1A**, clearly illustrating their interactions. Both compounds form a close interaction with the active site residue, His363 of SIRT1, along with several other interactions (**Fig 1B**). Both compounds bind into the deep binding cavity of SIRT1 with a good structural complementarity fit (**Fig 1C**). Examination of the docking positions of Doxercalciferol and Timiperone and their comparison with Selisistat implies that these compounds can tightly occupy the binding site of SIRT1. Overall, these compounds established robust interactions with crucial amino acid residues, indicating a plausible binding mode and potential inhibitory effects.

**Fig 2:**
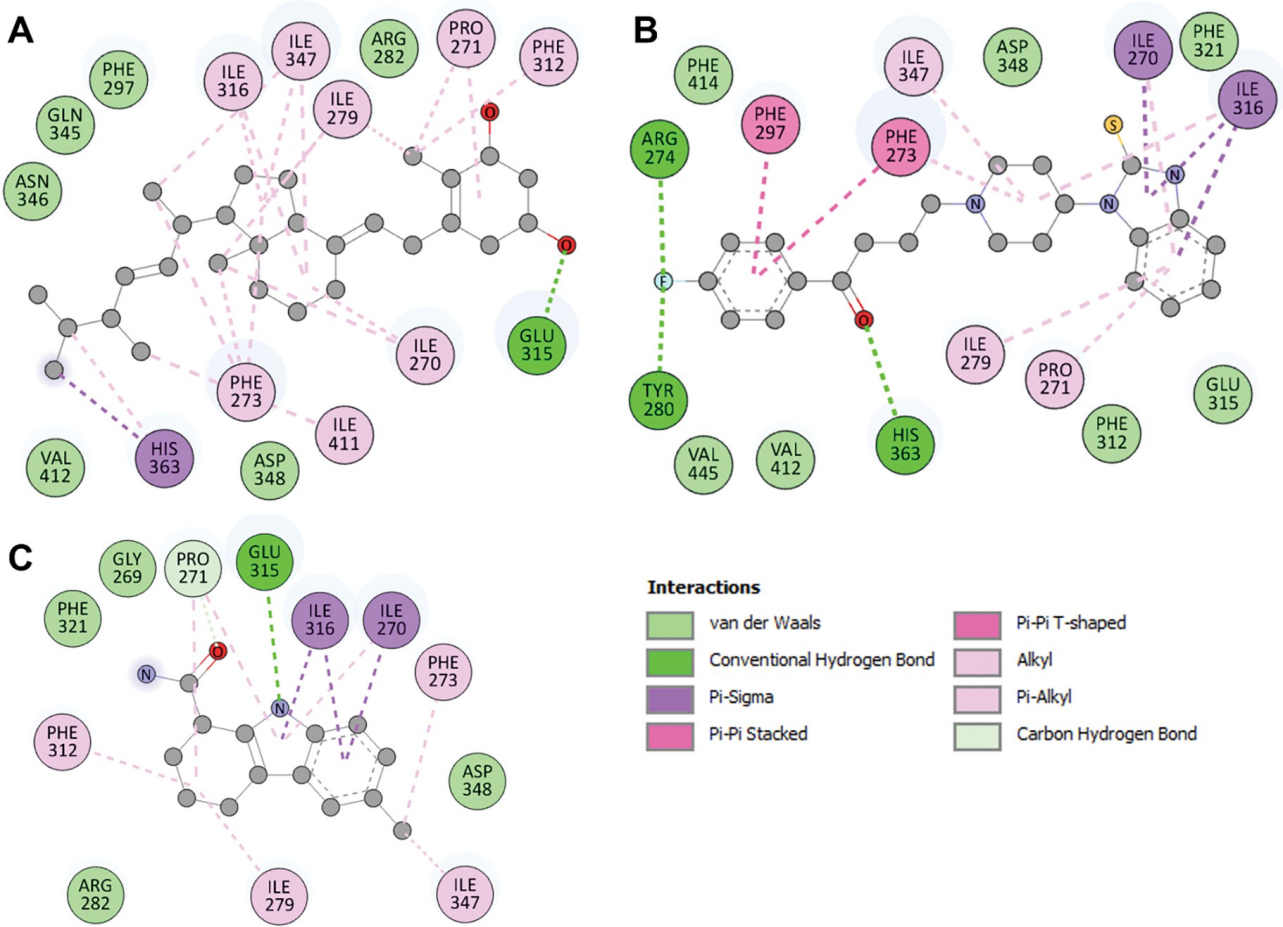
2D structural representation of SIRT1 residues interacting with (**A**) Doxercalciferol, (**B**) Timiperone, and (**C**) Selisistat.

These compounds that underwent docking within SIRT1’s binding pocket were thoroughly assessed for their interaction with the protein’s functionally significant residues. The types of interactions and involved amino acid residues with the chosen compounds are visually depicted in **Fig 2**. The interactions analysis revealed the common interaction of the selected compounds and the reference inhibitor Selisistat with the active site region of SIRT1. Notably, Doxercalciferol binds with the crucial residues within the active site pocket, including Glu315 and His363, along with several other important residues of the binding pocket (**Fig 2A**). At the same time, Timiperone forms three hydrogen bonds with Arg274, Tyr280, and His363, along with several other important residues of the SIRT1 binding pocket (**Fig 2B**). Both compounds showed several common interactions with the reference inhibitor Selisistat (**Fig 2C**). Based on these interactions and previous findings, the study suggested that the elucidated compounds might have the potential to inhibit SIRT1 activity [40].

### 3.3 PASS analysis: Biological activity predictions

The PASS analysis was carried out to explore all the potential biological properties of the shortlisted compounds relevant to SIRT1-associated complexities. The exploration of the biological activities of the selected compounds through the PASS analysis resulted in similar kinds of properties. Doxercalciferol and Timiperone underwent rigorous pharmacological evaluation to assess their potential as SIRT1 inhibitors. Both compounds demonstrated favorable drug-like properties, highlighting their potential for therapeutic application. Doxercalciferol and Timiperone have shown predictions for antipsoriatic, antiosteoporotic, bone diseases treatment, antieczematic, antineoplastic, antineurotic, antiischemic, antiacute neurologic disorders, antipsychotic, CNS active muscle relaxant activity with Pa ranging from 0,971 to 0,199 when Pa > Pi (**Table 2**). Higher Pa values indicate a higher likelihood that the compounds possess activity in the specific context or for a particular activity. The reference compound, Selisistat, demonstrated inhibitory activity against SIRT1, thereby validating the accuracy of our predicted results. This analysis supported the potential of elucidated compounds for therapeutic development against SIRT1-associated complexities.

**Table 2:** List of selected compounds and their biological properties calculated through PASS webserver. Pa, probability to be active; Pi, probability to be inactive.

S. No.	Compound	Pa	Pi	Biological Activity
1.	Doxercalciferol	0,971	0,002	Antipsoriatic
		0,968	0,003	Antiosteoporotic
		0,967	0,003	Bone diseases treatment
		0,954	0,002	Antieczematic
		0,922	0,005	Antineoplastic
2.	Timiperone	0,899	0,005	Antineurotic
		0,508	0,101	Antiischemic, cerebral
		0,478	0,095	Acute neurologic disorders treatment
		0,377	0,036	Antipsychotic
		0,199	0,053	CNS active muscle relaxant
3.	Selisistat	0,824	0,025	Phobic disorders treatment
		0,648	0,001	Sirtuin inhibitor
		0,630	0,062	Antineurotic
		0,425	0,006	Age-related macular degeneration treatment
		0,374	0,022	Angiogenesis stimulant

### 3.4 MD simulations

To gain insights into the dynamic behavior and stability of the SIRT1-Doxercalciferol and SIRT1-Timiperone complexes, all-atom MD simulations for 500 ns were performed. The trajectory analysis revealed that the complex maintains structural integrity and stability over the 500 ns simulation period. Various structural parameters for the protein and protein-ligand complexes were analyzed in a time-evolution manner as discussed in ensuing sections.

#### 3.4.1 Structural deviations and compactness

**Fig 3:**
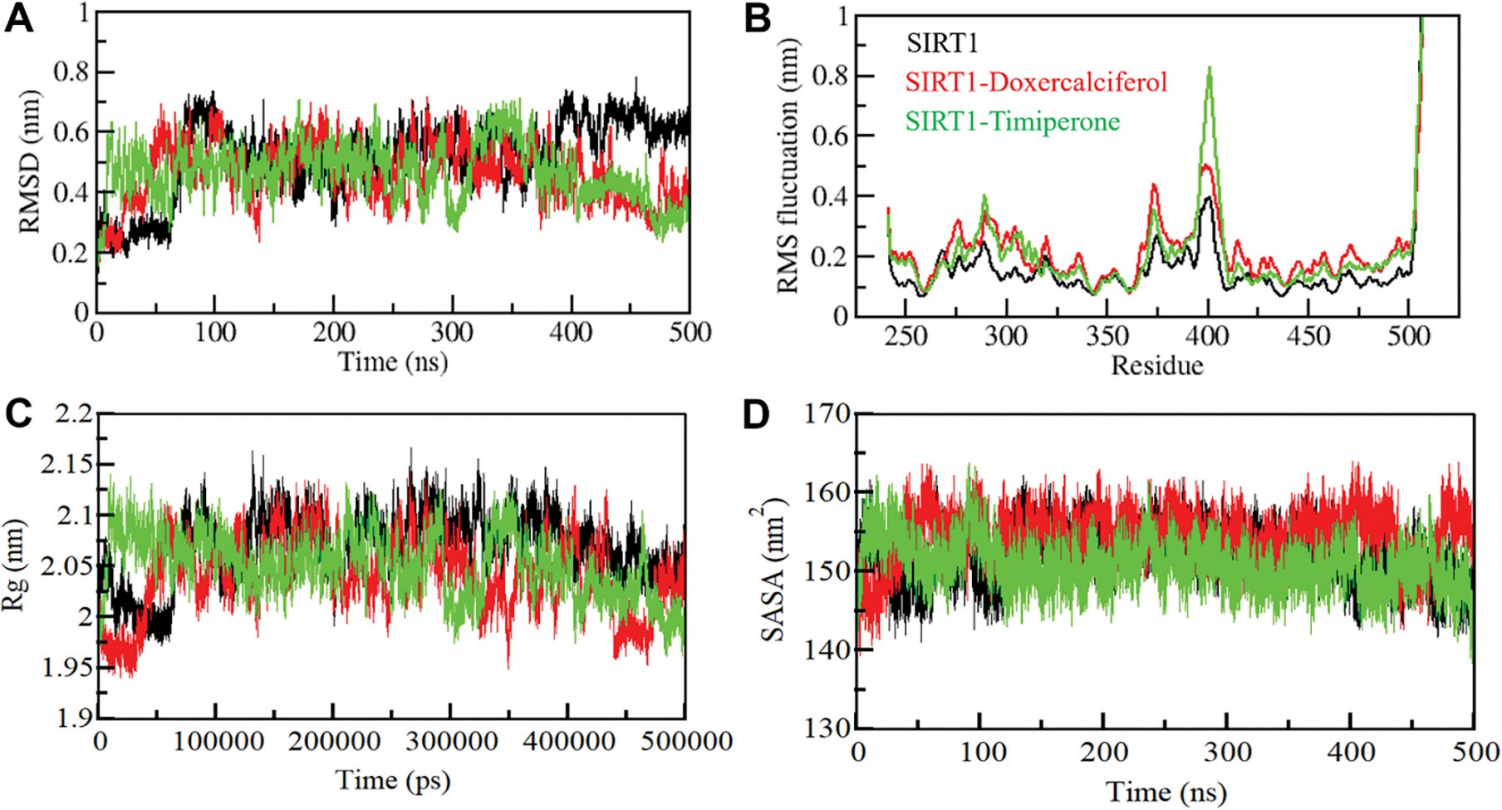
Structural dynamics and compactness of SIRT1 upon Doxercalciferol and Timiperone binding as a function of time. (**A**) RMSD plot of SIRT1 in complex with Doxercalciferol and Timiperone. (**B**) Residual fluctuations (RMSF) plot of SIRT1 before and after Doxercalciferol and Timiperone binding. (**C**) Time evolution of radius of gyration (*R*g). (**D**) SASA plot of SIRT1 as a function of time.

The assessment of structural variations and dynamics within a protein’s architecture has involved the application of the root-mean-square deviation (RMSD) metric [41]. Throughout the simulations, we tracked the time-dependent changes in RMSDs for SIRT1 and the SIRT1-Doxercalciferol and SIRT1-Timiperone complexes, yielding average values of 0.52 nm, 0.47 nm, and 0.47 nm, respectively. The average RMSD of the SIRT1-Doxercalciferol and SIRT1-Timiperone complexes are identical, achieving stability across the trajectory compared to unbound SIRT1. The resultant RMSD graph (**Fig 3A**) illustrates the stability of SIRT1 and the SIRT1-Doxercalciferol and SIRT1-Timiperone complexes over the simulation period. Nonetheless, various fluctuations reaching up to 0.7 nm are noticeable throughout. However, these fluctuations are minor which indicate a consistent and balanced RMSD pattern throughout the simulation.

To delve into the residual flexibility in free-state SIRT1 and its complexes upon Doxercalciferol and Timiperone binding, we computed the mean fluctuation of all residues and depicted it as the root-mean-square fluctuation (RMSF) plot (**Fig 3B**). The RMSF chart unveils diverse residual fluctuations within distinct regions of SIRT1. Notably, in the case of SIRT1-ligand, these fluctuations tend to follow the same pattern as free SIRT1 spanning from the N- to C-termini. The RMSF plot revealed remarkable stability in the fluctuations of RMSF following the binding of ligands, suggesting excellent stability within the protein-ligand system. However, upon binding with Timiperone, a slight increase in residual vibrations was observed in specific regions, indicating heightened fluctuations localized to certain loops. Consequently, the SIRT1-Timiperone complex seemed to exhibit greater stability compared to the SIRT1-Doxercalciferol complex, as corroborated by the RMSF analysis.

The conformational stability of SIRT1 was analyzed before and after the binding of Doxercalciferol and Timiperone, employing the calculation of the radius of gyration (*R*g) for both scenarios. The computed average *R*g values for SIRT1 in its unbound state and following Doxercalciferol and Timiperone binding were found to be between 2.95 nm to 2.15 nm. Analysis of the *R*g plot indicated negligible alterations in the folding of SIRT1 upon interaction with Doxercalciferol and Timiperone. An initial fluctuation in *R*g, observed up to 60 ns of MD trajectories, may be attributed to the initial packing adjustment of SIRT1. However, beyond this point, the *R*g values exhibited stability and equilibrium throughout the simulation, signifying the robustness of the complexes (**Fig 3C**).

Regarding the average solvent-accessible surface area (SASA) values, SIRT1 and the SIRT1-Doxercalciferol and SIRT1-Timiperone complexes displayed mean SASA values of 152.02 nm^2^, 154.34 nm^2^ and 150.88 nm^2^, respectively. The SASA plot exhibited a similar trend of equilibration for all three systems. A slight reduction in the average SASA values of SIRT1-Timiperone suggests a tighter packing of SIRT1 upon binding with Timiperone, possibly contributing to enhanced compactness and interaction (**Fig 3D**).

#### 3.4.2 Dynamics of hydrogen bonds

**Fig 4:**
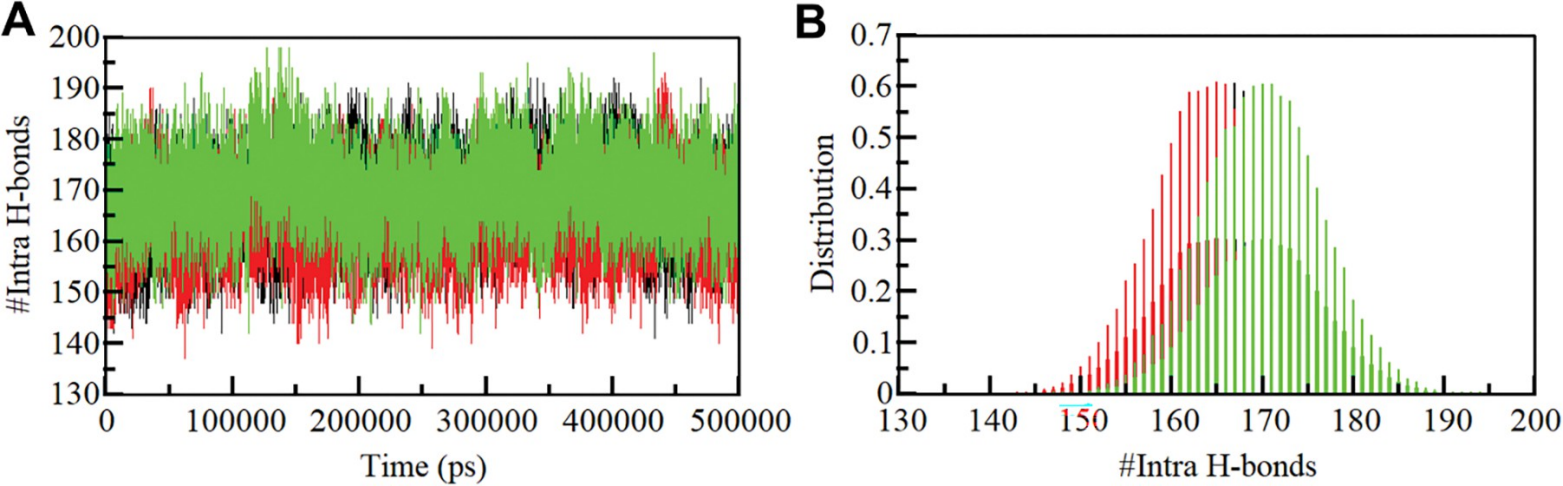
Intramolecular hydrogen bonding. (**A**) Time evolution and stability of hydrogen bonds (H-bonds) formed within 0.35 nm Intra-SIRT1 before and after Doxercalciferol and Timiperone binding. (**B**) The probability distribution function (PDF) of the H-Bonds for both the systems.

In order to gain a more comprehensive insight into the stability of SIRT1 before and after its interaction with Doxercalciferol and Timiperone, we meticulously examined the temporal evolution of hydrogen bonds formed within proximity of 0.35 nm during the simulation. Within the SIRT1 structure, the meticulous assessment revealed an average count of 167 intramolecular hydrogen bonds before Doxercalciferol and Timiperone binding, which slightly adjusted to 165 and 170 after the binding event (**Fig 4A**). To gain a better understanding of the hydrogen bond dynamics, we also constructed the probability distribution function (PDF) representing the occurrence of hydrogen bonds for both scenarios (**Fig 4B**).

**Fig 5:**
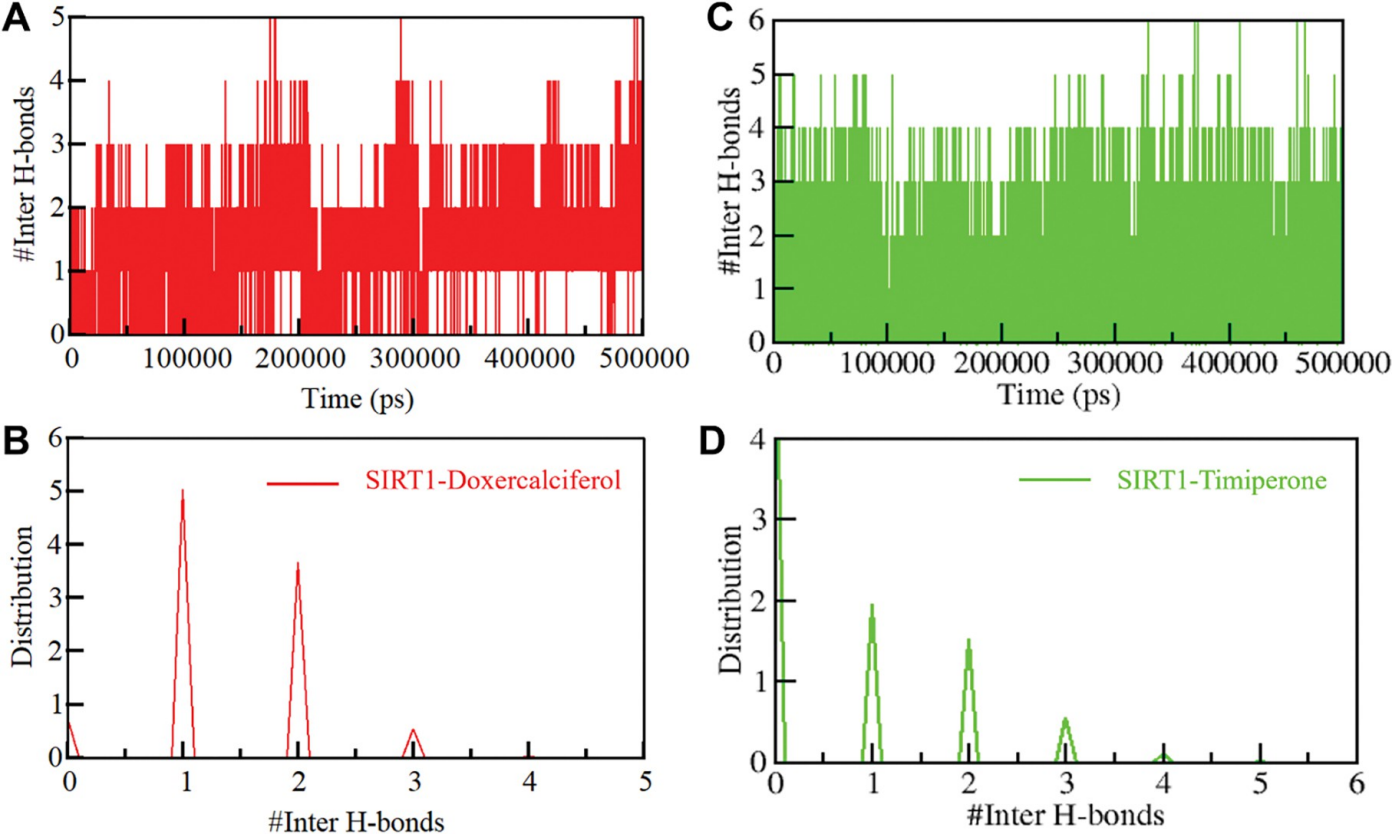
Intermolecular hydrogen bonding. (A) The time evolution plot shows the formation of intermolecular hydrogen bonds between SIRT1 and Doxercalciferol. (B) The plot shows PDF of SIRT1 and Doxercalciferol hydrogen bonds. (C) Time evolution plot of hydrogen bonds between SIRT1 and Timiperone. (D) PDF plot of hydrogen bonds between SIRT1 and Timiperone.

We also investigated the persistence of intermolecular hydrogen bonds in the protein-ligand complexes (**Fig 5**). The time-dependent formation of these bonds was analyzed using the *gmx hbond* utility of GROMACS. The study found that the SIRT1-Doxercalciferol and SIRT1-Timiperone complexes had an average of two hydrogen bonds, indicating stability and consistency over time (**Fig 5A** and **5C**). The PDF values also showed higher consistency of hydrogen bonds in the complexes (**Fig 5B** and **5D**). These results suggest that the hydrogen bonds play a crucial role in stabilizing the SIRT1-Doxercalciferol and SIRT1-Timiperone complexes and maintaining their stability. Additionally, the docking positions of Doxercalciferol and Timiperone were found to remain stable throughout the simulations, further supporting the stability of both complexes. Overall, these findings provide important insights into the molecular interactions that govern the protein-ligand binding and contribute to our understanding of stable complexes.

### 3.5 Secondary structure analysis

**Fig 6:**
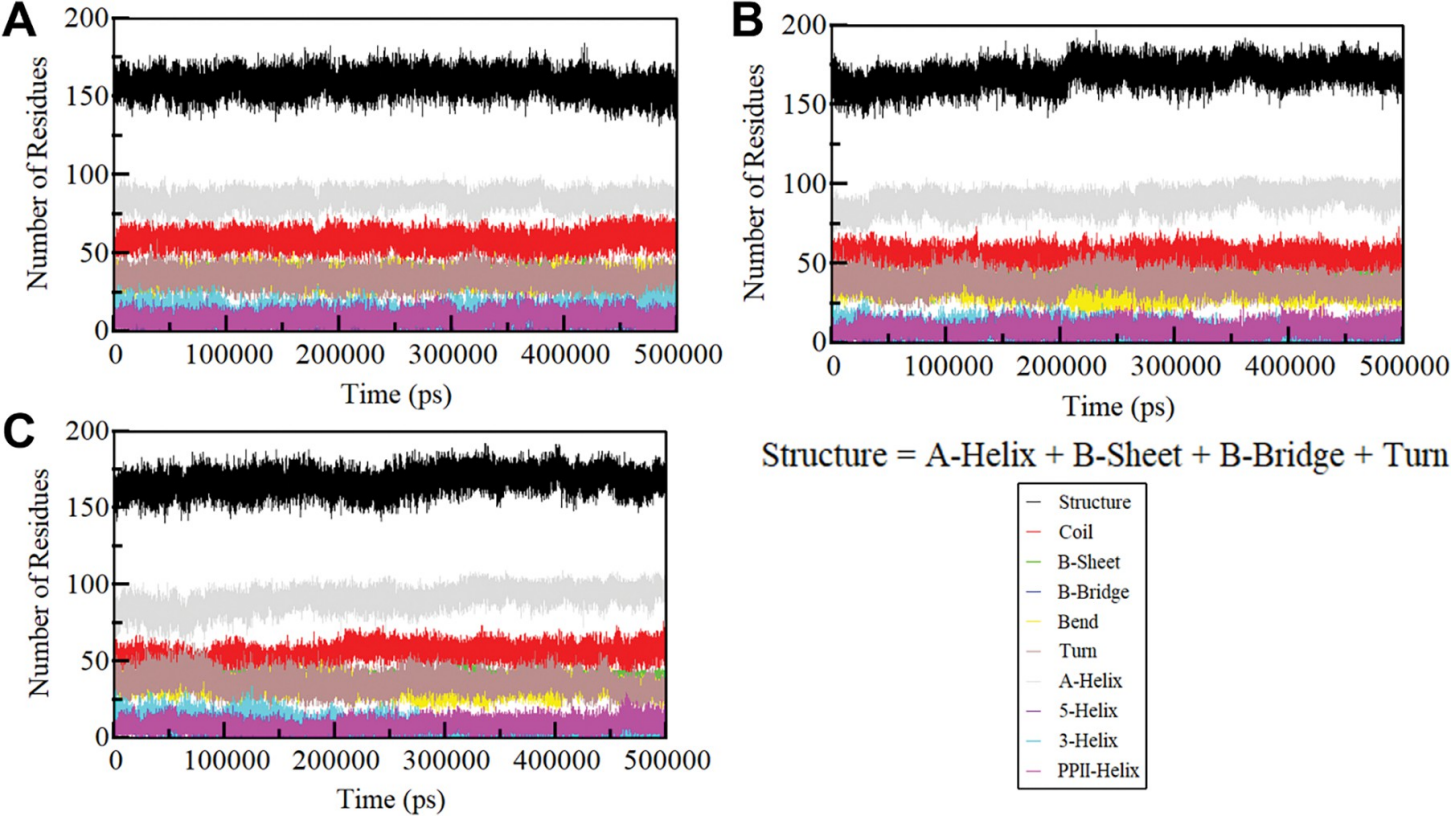
Secondary structure dynamics of (A) free SIRT1, (B) SIRT1-Doxercalciferol, and (C) SIRT1-Timiperone. The secondary structure content in SIRT1 is the sum of α-helix, β-sheet, β-bridge, and turns.

We further delved into the time-evolution of structural content to assess the effects of Doxercalciferol and Timiperone binding on the secondary structure of SIRT1 (**Fig 6**). The analysis demonstrated that the secondary structure composition of free SIRT1 remained notably constant throughout the simulation (**Fig 6A**). Similarly, the secondary structure makeup of SIRT1 remained quite stable after the binding of Doxercalciferol and Timiperone (**Fig 6B** and **6C**). This analysis suggests that the alterations in the secondary structure of SIRT1 following Doxercalciferol and Timiperone binding were relatively modest, underscoring the stability of the SIRT1-Doxercalciferol and SIRT1-Timiperone complexes. The preservation of these secondary structure elements further reinforces the stability and conservation of the native protein conformation in the presence of Doxercalciferol and Timiperone.

### 3.6 Principal component analysis

**Fig 7:**
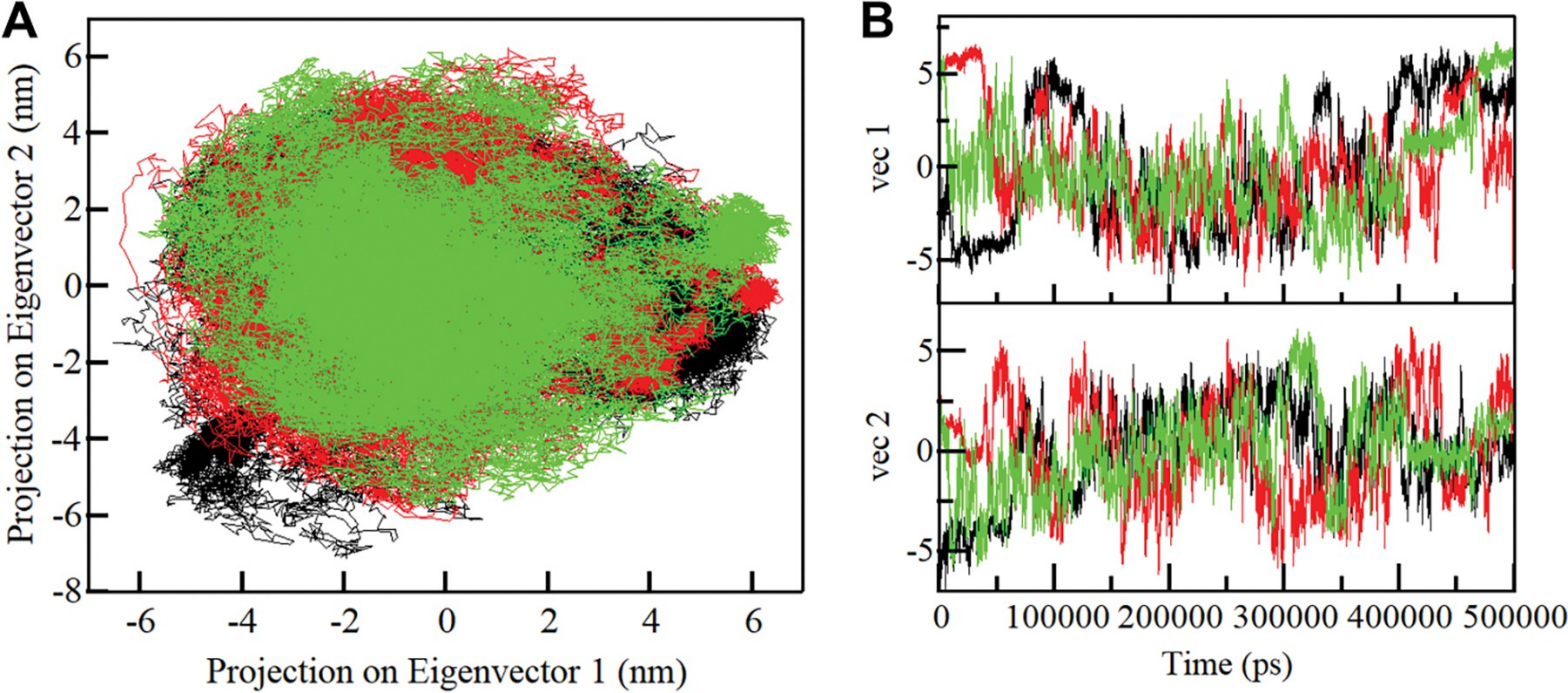
Principal component analysis. **(A)** 2D projections of trajectories on eigenvectors (EVs) showing conformational projections of SIRT1, SIRT1-Doxercalciferol and SIRT1-Timiperone (**B**) The time-evolution of projections of trajectories on both EVs.

PCA stands as an invaluable technique for discerning the predominant patterns within protein motions [42]. PCA facilitates a clearer understanding of its dynamics by illuminating the configurational space covering and the protein’s motion with a limited set of essential degrees of freedom. Our study also harnessed PCA to uncover the conformational exploration undertaken by free SIRT1, SIRT1-Doxercalciferol and SIRT1-Timiperone complexes. This effort involved investigating their collective motions through the essential dynamics approach. The intricate conformational sampling of the free SIRT1 and the SIRT1-Doxercalciferol and SIRT1-Timiperone complexes were effectively portrayed within the essential subspace, as visually depicted in **Fig 7A**. This projection illustrated the conformational range of SIRT1 in conjunction with the projection of its Eigen Vectors (EV-1 and EV-2) delineated by the protein’s Cα atoms. Our findings revealed that the SIRT1-Doxercalciferol and SIRT1-Timiperone complexes inhabited the same conformational subspace as free-state SIRT1. Although a marginal reduction was observed in both EVs for the complexes, this alteration did not prompt an overall shift in the underlying motion dynamics (**Fig 7B**).

### 3.7 Free energy landscape analysis

**Fig 8:**
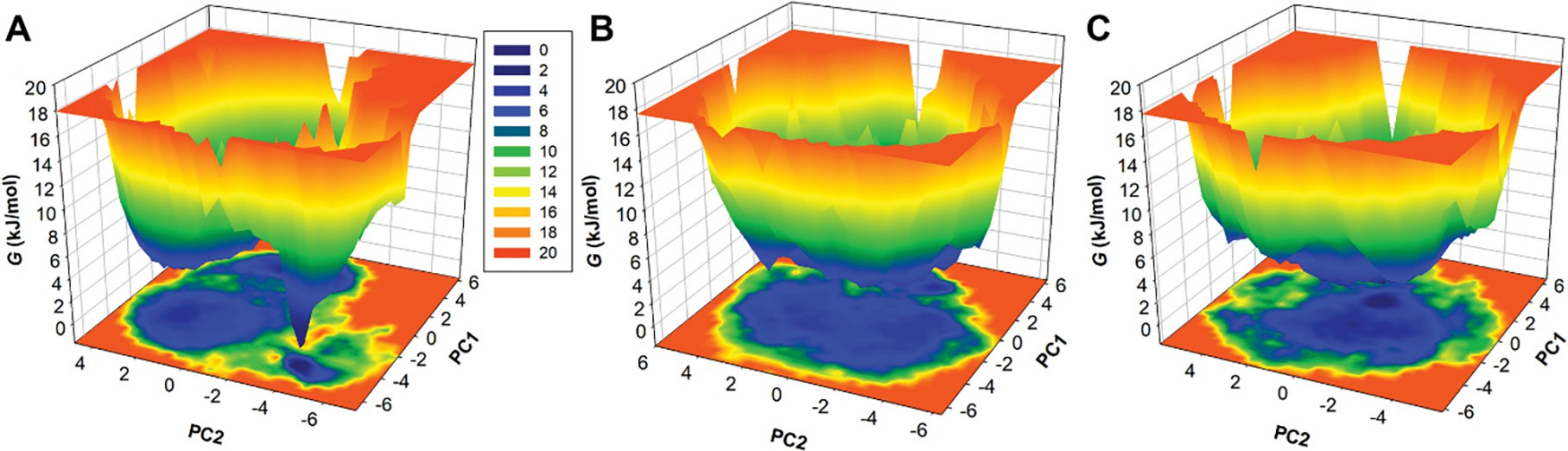
The Gibbs free energy landscapes for (A) free SIRT1 (B) SIRT1-Doxercalciferol and Timiperone.

The analysis of the FEL delivers a high-resolution understanding of stability of a protein-ligand binding system, unveiling potential binding transition states and metastable configurations [39]. This intricate information bears significant relevance in modern drug-discovery efforts, particularly in the design of inhibitors. To delve into the conformational stability and inherent states of free SIRT1, SIRT1-Doxercalciferol and SIRT1-Timiperone complexes, we constructed FELs employing the first two principal components (PCs). The contoured FELs for SIRT1, the SIRT1-Doxercalciferol and SIRT1-Timiperone complexes are graphically depicted in **Fig 8**. When examining these plots, the intensity of the color deepening to blue signifies lower energy states in proximity to the native configurations. The analysis of the FELs exposed distinctive insights. SIRT1 is characterized by a solitary global minimum encapsulated within three localized basins. Conversely, when Doxercalciferol and Timiperone interact with SIRT1, a large state emerges, housing to a large global minimum (**Fig 8B** and **8C**). This observation illustrates the presence of three distinct local basins, indicative of varying conformational dynamics. Collectively, the comprehensive evaluation of Doxercalciferol and Timiperone in MD simulation investigations indicates them as potential leads for drug development against SIRT1.

## 4. Discussion

The utilization of computational methodologies in the pursuit of lead discovery, particularly through the screening of large chemical libraries against predefined drug targets, has proven commendable [22]. A recent study employs an integrated computational approach that harnesses various machine learning techniques, including Inductive Logic Programming (ILP) and molecular docking, to sift through an extensive dataset of compounds sourced from Chinese herbs [43]. The objective of this study was to identify potential inhibitors of SIRT1. This study successfully pinpoints three promising compounds: ZINC08790006, ZINC08792229, and ZINC08792355, which exhibit noteworthy docking scores when interacting with the SIRT1 active site [43]. Importantly, the compounds elucidated in our study showed better docking scores compared to these proposed compounds.

In another study utilizing virtual screening, tipranavir emerges as a highly promising inhibitor of SIRT1, showcasing potent anti-hepatocarcinoma properties [44]. Remarkably, tipranavir demonstrates selective inhibition of HepG2 cell proliferation, while leaving normal human hepatic cells unharmed and devoid of toxicity. Additionally, tipranavir treatment leads to a substantial reduction in SIRT1 expression within HepG2 cells and triggers apoptosis. Importantly, in an *in vivo* xenograft mouse model, tipranavir effectively suppresses tumorigenesis and concurrently diminishes SIRT1 expression levels [44]. This robust evidence underscores the potential of tipranavir as a therapeutic agent for hepatocarcinoma. In yet another recent study centered around AI-powered virtual screening, novel SIRT1 inhibitors are uncovered [45]. This study highlights how AI-driven algorithms can efficiently narrow down extensive screening collections to target-specific sets comprising only a few hundred small molecules. This approach greatly accelerates and streamlines the process of discovering potential hits, offering a more cost-effective and rapid path to drug discovery. In summary, these findings unequivocally emphasize the valuable applications of computational approaches and their significant potential in advancing drug discovery, particularly in the context of SIRT1 targeting.

Here, we conducted a rigorous screening of a comprehensive library comprising FDA-approved compounds, with a particular focus on their binding potential and pharmacological attributes. Utilizing advanced molecular docking techniques, we were able to identify a specific group of compounds that exhibited exceptional binding affinity for SIRT1. A detailed analysis of their interactions uncovered a striking revelation: these chosen compounds effectively occupied a crucial active site pocket, which had previously been occupied by a majority of co-crystallized ligands [11, 40]. The SIRT1 active site, prominently featuring Glu315 and His363 residues, is pivotal for its functional activity [12]. This active site, present within the main catalytic core of the protein, governs the crucial functions of SIRT1 [11]. Among the screened compounds, our study highlighted two compounds, Doxercalciferol and Timiperone, that consistently interacted with the binding-site residues of SIRT1. Further exploration ensued through the application of PASS analysis, a tool wielded to unearth the biological properties of the compounds [36]. Drawing from the nexus of PASS analysis and the specific interactions observed with the SIRT1 active site, we discerned the emergence of Doxercalciferol and Timiperone as potent candidates in inhibiting SIRT1.

MD simulations are a powerful computational technique used in drug discovery to study the behavior of biological molecules at the atomic level [46]. They provide insights into the dynamic properties of proteins, which are crucial for understanding their function and interactions with potential drug compounds [47]. Here, RMSD analysis supported the equilibrium in RMSD over a 500 ns simulation period, demonstrating the stability of the SIRT1-Doxercalciferol and SIRT1-Timiperone complexes. A detailed analysis was performed via RMSF fluctuations, tracing the fluctuations of each residue in the backbone of SIRT1 before and after Doxercalciferol and Timiperone binding. These fluctuations demonstrated stability, abating significantly upon Doxercalciferol and Timiperone binding, an example of the robustness within the protein-ligand complex. The trajectory of *R*g and SASA illustrated SIRT1’s stability with Doxercalciferol and Timiperone under solvent conditions. The arrangement of intramolecular and intermolecular hydrogen bonds further probed this aspect and revealed that the number of hydrogen bonds within SIRT1 and its complexes remained stable during the simulation.

The structural dynamics and conformational exploration were further explored by evaluating the secondary structure dynamics and phase space performance. Visualizing the conformational sampling of SIRT1-Doxercalciferol and SIRT1-Timiperone, compared with EV-1 and EV-2 projected by the protein C_α_ atoms, yielded an overlapping leap with the stable clusters of the free SIRT1. Parallel, FEL plots indicated a minor transition in the complexes, as the binding of Doxercalciferol and Timiperone to SIRT1 caused variations in the dimensions and coordinates of the sampled essential subspace, though strongly settled within a confined global minimum. Overall, we can say that computational methodologies play a pivotal role in modern drug discovery. The enlightening findings suggest that Doxercalciferol and Timiperone can be effective molecules for developing potential SIRT1 inhibitors, offering possibilities for addressing various complex diseases like cancer, neurodegenerative disorders, and metabolic syndromes.

## 5. Conclusion

This study elucidates the promising potential of targeting SIRT1 as a viable therapeutic strategy, focusing on developing effective inhibitors. Through a systematic structure-based drug discovery approach, we identified two compounds, Doxercalciferol and Timiperone, which exhibit remarkable SIRT1-binding properties. The findings achieved in this study are underscored by the substantial improvements in pharmacological attributes, binding affinity, and exceptional stability exhibited by Doxercalciferol and Timiperone in their interaction with SIRT1. The comprehensive MD simulation study further reinforces the credibility of our findings, revealing the formation of a robust and stable complex between SIRT1-Doxercalciferol and SIRT1-Timiperone. These collective outcomes corroborate the notion that Doxercalciferol and Timiperone possess the required characteristics to serve as potent compounds for the development of SIRT1 targeting therapeutic strategies.

The implications of our findings extend to a wide array of debilitating conditions, including cancer, neurodegenerative disorders, and metabolic syndromes. The observed SIRT1-inhibitory potential of Doxercalciferol and Timiperone positions it as a prospective candidate for therapeutic interventions in these critical medical fields. However, it is crucial to emphasize that further rigorous clinical validations are necessary before Doxercalciferol and Timiperone can move into clinical applications. In summary, our study sheds light on the exciting potential of Doxercalciferol and Timiperone as lead compounds and underscores the significance of a structure-based drug discovery approach. The combination of molecular insights and computational simulations has paved the way for a promising avenue in SIRT1-targeted therapeutics. As we look ahead, our work sets the stage for further investigations and refinements, culminating in the development of innovative treatments that can potentially ease the burden of devastating diseases on global health.

## Supporting information

S1 Fig Binding pose of Selisistat analogue in complex with SIRT1. Superimposed yellow and cyan elements show co-crystallized and docked Selisistat analogue with SIRT1 in green, respectively. Co-crystallized SIRT1-Selisistat analogue complex coordinates were downloaded from RCSB-Protein Data Bank with PDB ID: 4I5I.
(DOCX)
