# Supplementary material for: Targeting Sirtuin 1 for therapeutic potential: Drug repurposing approach integrating docking and molecular dynamics simulations
Source: PLoS One. 2023 Dec 20;18(12):e0293185. doi: 10.1371/journal.pone.0293185 (PMC10732437; doi:10.1371/journal.pone.0293185)
Supplement: S1 Fig — Superimposed yellow and cyan elements show co-crystallized and docked Selisistat analogue with SIRT1 in green, respectively. Co-crystallized SIRT1-Selisistat analogue complex coordinates were downloaded from RCSB-Protein Data Bank with PDB ID: 4I5I. (DOCX) [file pone.0293185.s001.docx]

*Supplementary File*

**Targeting Sirtuin 1 for therapeutic potential: Drug repurposing approach integrating docking and molecular dynamics simulations**

Mohammed Alrouji^1^, Fahad A Alhumaydhi^2^, Abdulrhman Alsayari^3,4^, Sharaf E Sharaf^5^, Sheeba Shafi^6^, Saleha Anwar^7^, Moyad Shahwan^8^, Akhtar Atiya^3#^, Anas Shamsi^8*^

*^1^Department of Medical Laboratories, College of Applied Medical Sciences, Shaqra University, Shaqra 11961, Saudi Arabia. Email:* [*malrouji@su.edu.sa*](mailto:malrouji@su.edu.sa)

*^2^Department of Medical Laboratories, College of Applied Medical Sciences, Qassim University,*

*Buraydah 52571, Saudi Arabia. Email:* [*f.alhumaydhi@qu.edu.sa*](https://ajman4-my.sharepoint.com/personal/m_shamsi_ajman_ac_ae/Documents/New%20Tasks/Manuscripts%20submitted/SIRT1_PlosOne/f.alhumaydhi@qu.edu.sa)

*^3^Department of Pharmacognosy, College of Pharmacy, King Khalid University (KKU), Guraiger St., Abha 62529, Saudi Arabia. Email:* [*atkhan@kku.edu.sa*](https://ajman4-my.sharepoint.com/personal/m_shamsi_ajman_ac_ae/Documents/New%20Tasks/Manuscripts%20submitted/SIRT1_PlosOne/atkhan@kku.edu.sa)

*^4^Complementary and Alternative Medicine Unit, King Khalid University (KKU), Abha 62529, Saudi Arabia. Email:* [*alsayari@kku.edu.sa*](mailto:alsayari@kku.edu.sa)

*^5^Pharmaceutical Chemistry Department, College of Pharmacy Umm Al-Qura University Makkah - Saudi Arabia. Email:* [*sesharaf@uqu.edu.sa*](https://ajman4-my.sharepoint.com/personal/m_shamsi_ajman_ac_ae/Documents/New%20Tasks/Manuscripts%20submitted/SIRT1_PlosOne/sesharaf@uqu.edu.sa)

*^6^Centre for Interdisciplinary Research in Basic Sciences, Jamia Millia Islamia, Jamia Nagar, New Delhi 110025, India. Email:* [*email2saleha@gmail.com*](file:///\\tsclient\WebFile\email2saleha@gmail.com)

*^7^Department of Nursing, College of Applied medical sciences, King Faisal university, Al- Ahsa, 31982. Email:* [*sheeba@kfu.edu.sa*](mailto:sheeba@kfu.edu.sa)

*^7^Center for Medical and Bio-Allied Health Sciences, Ajman University, UAE. Email:* [*anas.shamsi18@gmail.com*](mailto:anas.shamsi18@gmail.com)*;* [*moyad76@hotmail.com*](mailto:moyad76@hotmail.com)

***Corresponding Author***

***Anas Shamsi, PhD**

Centre of Medical and Bio-allied Health Sciences Research

Ajman University

United Arab Emirates.

Email: [anas.shamsi18@gmail.com](mailto:anas.shamsi18@gmail.com)

***#Co Corresponding Author***

Akhtar Atiya

Department of Pharmacognosy

College of Pharmacy

King Khalid University (KKU), Guraiger St., Abha 62529, Saudi Arabia.

*Email:* [*atkhan@kku.edu.sa*](https://ajman4-my.sharepoint.com/personal/m_shamsi_ajman_ac_ae/Documents/New%20Tasks/Manuscripts%20submitted/SIRT1_PlosOne/atkhan@kku.edu.sa)


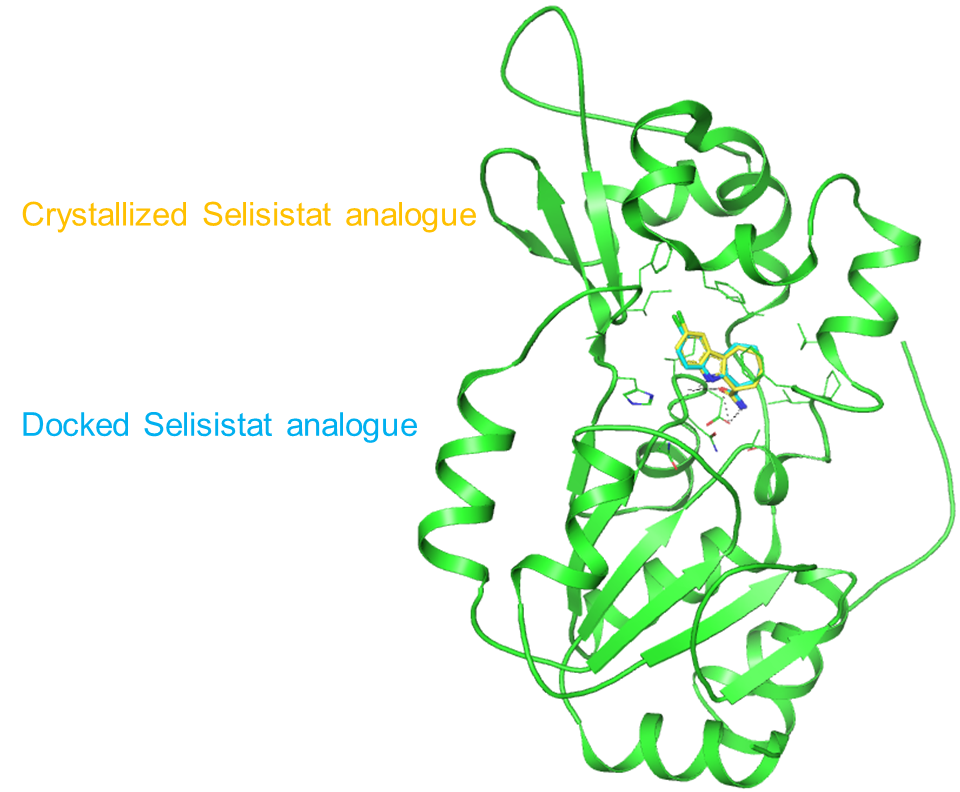


**Figure S1:** Binding pose of Selisistat analogue in complex with SIRT1. Superimposed yellow and cyan elements show co-crystallized and docked Selisistat analogue with SIRT1 in green, respectively. Co-crystallized SIRT1-Selisistat analogue complex coordinates were downloaded from RCSB-Protein Data Bank with PDB ID: 4I5I.
